# Volatile Organic Compounds Emitted by Flowers: Ecological Roles, Production by Plants, Extraction, and Identification

**DOI:** 10.3390/plants13030417

**Published:** 2024-01-31

**Authors:** Mame-Marietou Lo, Zohra Benfodda, Roland Molinié, Patrick Meffre

**Affiliations:** 1 UPR Détection, Évaluation, Gestion des Risques CHROniques et éMErgents (CHROME), UNIV. NIMES, CEDEX 1, F-30021 Nîmes, France; lmarietou@outlook.fr (M.-M.L.); zohra.benfodda@unimes.fr (Z.B.); 2 UMR INRAE 1158 Transfrontaliére BioEcoAgro, BIOlogie des Plantes et Innovation (BIOPI), UPJV, UFR de Pharmacie, F-80037 Amiens, France; roland.molinie@u-picardie.fr

**Keywords:** volatile organic compounds (VOCs), flowers, plant ecology, isolation methods, identification methods

## Abstract

Volatile organic compounds (VOCs) with a large chemical diversity are emitted by plant flowers. These compounds play an important role in the ecology of plants. This review presents the different ecological roles of VOCs present in the odor plumes of plant flowers, such as pollination, defense, adaptation to their environment, and communication with other organisms. The production and accumulation sites of VOCs in plants with their spatial and temporal variations, including environmental issues, are also summarized. To evaluate the qualitative and quantitative chemical composition of VOCs, several methods of extraction and analysis were used. Headspace (HS) sampling coupled with solid phase microextraction (SPME) is now well-developed for the extraction process. Parameters are known, and several fibers are now available to optimize this extraction. Most of the time, SPME is coupled with gas chromatography-mass spectrometry (GC-MS) to determine the structural identification of the VOCs, paying attention to the use of several complementary methods for identification like the use of databases, retention indices, and, when available, comparison with authentic standards analyses. The development of the knowledge on VOCs emitted by flowers is of great importance for plant ecology in the context of environmental and climate changes.

## 1. Introduction

Volatile organic compounds (VOCs) are generally lipophilic compounds with low molecular weights and high vapor pressures. They represent about 1% of the secondary metabolites identified in the plant kingdom. Indeed, over 1700 volatile compounds have been described in more than 90 plant families [1,2,3]. They are produced in all plant organs such as roots, stems, leaves, fruits, seeds, as well as flowers, which have been found to release the greatest quantities and diversity of VOCs. Their physical properties allow them to cross cell membranes and be released into the atmosphere or soil in the absence of a diffusion barrier [4]. Depending on their biosynthetic pathway, floral VOCs can be classified into three major families: terpenoids, phenylpropanoids/benzenoids, and fatty acid and amino acid derivatives [1,5]. In addition to these, through specific biosynthetic pathways about which little information is available, compounds containing sulfur and nitrogen contribute to the attraction of pollinators to flowers [6]. The terpenoids form the largest group of VOCs, which are derived from the mevalonic acid (MVA) and the methylerythritol phosphate (MEP) pathways. Indeed, isoprenes, monoterpenes, and diterpenes are biosynthesized in the plastids by the MEP pathway, and sesquiterpenes are biosynthesized in the cytosol by the MVA pathway [7,8,9,10]. Phenylpropanoids/benzenoids are most often obtained by the phenylalanine pathway in the cytosol. This class is divided into three subgroups, according to their carbon skeleton structure: phenylpropanoids with a C6 to C3 skeleton, benzenoids with a C6 to C1 skeleton, and phenylpropanoid-related compounds with a C6 to C2 skeleton [11,12]. The third family of floral VOCs consisting of fatty acid derivatives are biosynthesized in chloroplasts and are derived from the C18 unsaturated fatty acids, linoleic acid, and linolenic acid [13,14]. Although they have attracted human attention since antiquity for their great utility in perfumery, cosmetics, aroma, and medicinal applications, the primary functions of floral volatiles are ecological [15]. Indeed, they mediate ecological interactions between flowers and a wide range of visitors, including pollinators, florivores, and pathogens, and also serve as signals in plant-to-plant communication [16]. Table 1 shows some examples of such ecological bioactivities with the structure of the associated VOC. However, it is possible that some VOCs have no function of their own and are merely by-products of necessary metabolic processes [17]. Depending on the species and under the influence of biotic and abiotic factors, the diversity and quantity of volatiles emitted by flowers may vary.

## 2. Functions of Floral Volatile Organic Compounds

### 2.1. Role of VOCs in Attracting Pollinators

Pollination is a very important biological process in the reproduction of higher plants that involves the transfer of pollen from male to female reproductive organs [19,40]. It is called self-pollination when this transfer can take place without the help of any other organism. However, the phenomenon of self-incompatibility is very often encountered, so plants resort to cross-pollination strategies that can be achieved by wind, insects, birds, and mammals such as bats. In the case of pollination by other living organisms, plants develop several interesting strategies to attract them [19]. In addition to visual and thermal signals, flowering species release a mixture of volatile compounds to attract pollinators [41] and seed dispersers for reproductive and evolutionary success [42]. This multisensory input (visual, olfactory, thermal) is essential for pollinators to locate food and reproductive sites [14]. Depending on the quantity, composition, and context of their release, volatile compounds convey information that elicits distinct behavioral responses in the respective pollinators. Indeed, for flowers emitting at night, the production of volatiles must be of high intensity to compensate for the reduced visibility of the flowers under low illumination. Also, the emission of volatiles is performed at long distance and contributes mainly to guide pollinators such as moths. For landing, feeding and breeding behaviors, these are triggered by the volatile compounds emitted over short distances [6]. The composition of the floral odor is also a very important element and allows the attraction of specific pollinators. For example, flies and beetles are attracted to floral odors similar to ‘rotten meat’ due to compounds such as indole or derivatives like skatole [38,39]. Some flowering plants, such as Orchids, are able to mimic the pheromones (sex hormones) of pollinating insects through the floral odor they emit, in addition to mimicking the shape and morphology of female insects so that “pseudo-copulation” and pollination occurs [19]. Other plants emit volatile compounds containing sulfur to attract necrophagous, saprophagous, and coprophagous insects [6]. In addition to attracting pollinators to flowers and guiding them to food resources within the flower, floral volatiles are essential for pollinators to distinguish between plant species and even between flowers of the same species. Indeed, while flowers may have identical shapes or colors due to the great diversity of volatile compounds, their relative abundances, and their interactions within the olfactory bouquet, no two floral scents are exactly alike. Thus, pollinators may use floral odor to discriminate a particular flower for which nectar or pollen is the reward [1].

### 2.2. Role in the Defense of the Plant

Emission and perception of plants volatiles are key points in plant fitness, including defense, attraction of pollinators, and also plant-plant, plant-bacteria, plant-animal signaling and responses to biotic stresses [43]. In response to external damage and attack, flowers emit a mixture of volatile compounds as a means of protecting their reproductive structures. VOCs provide both direct protection, by inhibiting or repelling aggressors. VOCs can also provide indirect protection, by attracting predators of the attacking florivores themselves such as parasitic wasps, flies, or predatory mites to protect the signal plant from further damage [26,44,45] or by interacting with bacterial colonization therefore interfering with plan-animal interactions [46]. However, it should be noted that these emitted volatiles are not always beneficial because they may attract other non-specific florivores resulting in increased attacks [21]. Flowers can also drastically reduce their VOC emission after pollination when pollinators also act as floral antagonists. For example, *Silene latifolia* flowers emit floral volatiles to attract the moth *Hadena bicruris*, however the moth larva feeding destroys about a quarter of the developing seeds, which has a detrimental effect on plant health. After pollination, the flowers strongly reduce the emission of the main VOCs to minimize further parasitism [47]. In other species such as *Petunia*, to defend themselves, the flowers specifically release deterrent volatiles rather than reducing VOC emission [48]. Thus, the complex mixtures of floral volatiles in these species are likely defined by selection pressure that arises from the need to maximize pollinator attraction while avoiding the damages caused by florivores.

Microorganisms colonize surface plant parts including flowers (anthosphere) and VOCs emitted by plants have an effect on these microbial colonizers including bacteria [46]. In addition to florivores, flowers produce VOCs with antimicrobial and antifungal properties to control pathogens [33]. Indeed, it was shown in *Arabidopsis thaliana* that flowers of plant lines lacking (*E*)-β-caryophyllene emission showed greater bacterial growth on their stigmas, and their seeds were lighter and deformed than those of plant lines with (*E*)-β-caryophyllene emission. The latter were more resistant and had reduced cell damage and higher seed production. Thus, β-caryophyllene is a volatile compound that inhibits bacterial growth and acts in defense against pathogens [30]. Also, a study conducted on the species *Saponaria officinalis* and *Lotus corniculatus* showed a much lower bacterial diversity on the flower petals than on the leaves of these plants, suggesting that antibacterial floral volatiles are the cause of this low bacterial diversity [49]. VOCs also play a role in the mechanism of response to abiotic stresses, especially in the context of drought, flooding, variation in temperature, light intensity, humidity, and other abiotic factors in the environment [50] that can be important in the climate changes context [43]. In this case, a stress messenger is released, which allows the plant to trigger defense mechanisms [14].

### 2.3. Role in Communication with Other Plants

Community life in the plant world requires very sophisticated means of interaction. VOCs represent a rapid and reliable means of communication for plants to interact with each other [51]. Plants have, therefore, adapted a variety of strategies to rapidly exchange volatile messages and reduce the impact of complex biotic and abiotic factors in their environment [52]. Depending on the context in which VOCs are emitted, the effectiveness of communication between neighboring plants is determined by the plants involved. The emitting plant encounters a specific stress and emits a unique mixture of volatiles that will trigger the activation or suppression of different genetically encoded programs (known as ‘priming’) in the receiving plants to prepare a response that will match the upcoming stress [53,54]. Indeed, plants undergoing florivore attack can signal the presence of danger to neighboring plants through the emission of volatile compounds. The receiving plants are thus prepared for an enhanced defense after receiving distress signals via VOCs from emitting plants. They will, therefore, be better protected in a floristic pressure environment, without having to suffer from costly energetic investments in defense mechanisms [14]. VOC emissions also provide valuable information about the reproductive environment of the plant. They allow the recipient plant to know whether neighboring plants are anthesizing, whether they are heterospecific or conspecific, and whether pollinators are present [55]. It has also been proposed that floral volatiles are used by conspecific neighbors to synchronize flowering because of their highly specific and informative value [56].

However, interactions through volatile compounds are not always positive, plants can have phytotoxic effects on their neighbors [57]. The volatiles released by the emitting plants can affect the growth or the state of flowering of the receiving plant, that is why these compounds are commonly used in plant competition. For example, it has been shown that floral volatiles from the snapdragon *Antirrhinum majus* have negative allelopathic effects on *Arabidopsis thaliana*. Indeed, they inhibit the growth of the roots of the latter and the compound responsible is methyl benzoate [31]. This shows that coexistence with other plants is one of the most important factors affecting the growth of plant individuals and the distribution of species. Thus allelopathic interactions are an important factor in determining the distribution and abundance of species within plant communities, and are also considered important in the success of many invasive plants [57]. Thus, VOCs carry information that can have a multitude of effects ranging from beneficial adaptations to plant competition and phytotoxicity. Volatile communication between plants is very complex, and small changes in the composition of the volatile mixture can have great significance for neighboring plants.

## 3. Formation and Accumulation Site

Volatile organic compounds are produced and accumulated in different structures more or less complex depending on the plant species. In most vascular plants, volatiles such as monoterpenes and sesquiterpenes are synthesized and stored in specific secretory tissues. These may be located on the surface of the plant, as trichomes, or within the plant and consist of single cells or groups of cells of different sizes. The secreted material is usually released from the secretory tissues outside the plant or into specialized intercellular spaces [58]. In some plant species, VOCs are accumulated within resin ducts or glandular trichomes and may be released in large quantities once these structures are disrupted by herbivore feeding or by movement on the plant surface [59]. As for the volatiles involved in defense, they can be toxic to the plant itself. The latter must therefore be able to store and release them without being poisoned, and they are therefore stored either in the form of inactive precursors, or example in the form of glycosides, or in extracellular compartments, as in the case of glandular trichomes [60]. Glandular trichomes are present in several plant families and their morphology can vary from one family to another. In fact, there are two types of secretory trichomes: peltate trichomes and caped trichomes. The first are composed of a basal epidermal cell, a short pedunculated cell and a secreting head cell made up of several secreting cells arranged in a single layer, and the capped trichomes are made up of a basal cell, one or more pedunculated cells and one or more secreting cells. VOCs are thus accumulated under the cuticle of these head cells [14]. Recently, it has been shown that cuticle is not only a passive diffusion barrier but plays an important role in VOC mass transfer being then an important member of the overall VOC network [61]. It has also been demonstrated that cell-wall non-specific lipid transfer facilitates emission of floral volatiles [62]. Other secretory structures producing VOCs involved in defense are less visible than trichomes, as they are hidden in the deep tissues of the plant. These are secretory ducts and cavities that consist of relatively large intercellular spaces lined by an epithelium of secretory cells [58].

In flowers, the biosynthesis of VOCs is generally performed at the epidermal cell level which allows them to easily escape into the atmosphere. In general, petals are the main source of floral volatiles, although other tissues (stamens, pistils, sepals, and nectaries) also contribute to the floral bouquet in some plant species [6]. The floral fragrance is indeed produced in osmophores or conical cells located on the petals. Osmophores are found throughout the inflorescence at the perianth, bracts, peduncle appendages, or anthers. Although different in shape, osmophores share common characteristics, i.e., they are usually oriented towards the adaxial side of the perianth and have a bullous, rough, hairy, conical, or papillary epidermis. Below the osmophores are several cell layers, forming the glandular tissue, which merge into the normal cell layers of the parenchyma [63]. Although osmophores are predestined for the production and emission of volatiles, they are not always necessary for the emission of fragrances. Indeed, when the multilayered production and release tissues present in osmophores are absent, the adaxial epidermis often has delicate conical bubbled cells to facilitate volatile emission [64]. For cone cells, their characterization and involvement in VOC secretion is well-characterized in the rose, which is an excellent model for studying petal secretory cells [65].

## 4. Spatial and Temporal Variation of VOCs

The floral scent emitted by plants over the life of the flower varies qualitatively and quantitatively for many reasons [66]. Moreover, odorless flowers for humans also emit VOCs [67]. To maximize pollinator attraction, floral fragrance emission is restricted to particular floral tissues and is regulated through development and timing [6]. In flowers, the petals are the primary emitters of volatiles, although various other parts of the flower may also participate in the emission of volatiles [68]. While the same floral odorant compounds are often emitted from all parts of the flower, they are not necessarily emitted in the same amounts, and in some cases, specific compounds are emitted from specific floral organs such as osmophores or floral glandular trichomes [19]. Emission from petals often serves to attract pollinators over long distances, while production in nectaries or pollen signals the availability of food sources. Tissue specificity of odor delivery is controlled at the level of odor biosynthetic gene expression and enzyme activity. Emission levels, corresponding enzyme activities, and the expression of genes encoding odor biosynthetic enzymes are all regulated in time and space during flower development. In general, the expression of odorant genes is relatively uniform, with the highest levels found in the petals [69]. With respect to the rhythmicity of floral odor, it is often correlated with the activity of the respective pollinators allowing plants to conserve energy and carbon at times when pollinators are inactive. For example, flowers that are pollinated by nocturnal insects such as moths tend to have maximum scent emission in the early evening, whereas diurnal rhythmicity has been observed in plants pollinated during the day [42].

The emission of floral scent often changes over the life span of flowers in addition to being spatially and rhythmically regulated. Indeed, emission levels are usually highest when flowers are ready for pollination, i.e., when anthers are dehiscent, and decrease during senescence. The level of volatiles emitted is reduced once the flower is pollinated to prevent further visits that might damage the flower but also to redirect visitors to the remaining unpollinated flowers [4,70]. In addition to this, environmental factors play an important role in the variation of floral emissions and there may be interactions between the typical temporal rhythms of a plant’s floral volatile production and composition and the environmental conditions it experiences. For example, recent studies show that anthropogenic air pollutants like nitrogen oxides or ozone, even at levels lower than what is considered environmentally safe, can have an impact on pollinator foraging [71]. However, this aspect is still poorly understood [72].

It is also interesting to note the interspecific variabilities of floral VOCs. The production and emission of VOCs are influenced qualitatively and quantitatively by genetic variability. Volatile profiles are indeed different from one plant species to another, some VOCs are emitted by the majority of species while others are specifically emitted by certain species or by plants belonging to certain families or genera [73]. As with intraspecific variation in floral VOCs, it is also likely that environmental conditions create variation in floral volatiles between species [72].

## 5. Extraction and Analysis of Floral VOCs

For all methods of analysis of plant volatiles, identification of the authentic profile of the mixtures of volatiles emitted by the plant is very important. However, the choice of which system to use in a particular experiment for the collection and analysis of plant volatiles generally depends on the biological problem and the plant material being studied. Collection of volatiles is easiest when performed in situ from whole plants. However, when we are specifically interested in volatiles emitted from reproductive organs or vegetative tissues, or when we want to determine whether stress-induced VOC emissions are local or systemic responses, or when we want to correlate VOC emissions with tissue-specific enzyme activities, it is often necessary to sample VOCs from plant parts or organs [74]. In this case, VOCs are sampled either from detached plant parts or preferably in situ from enclosed plant organs to avoid additional VOC emission due to wounding effects. In addition, the researcher must decide whether to be interested in a qualitative profile of volatiles released at a specific time or to measure quantitative changes in VOC emissions related to development or stress. In both cases, adequate instrumentation is required for the collection and analysis of volatiles.

Table 2 summarizes the methods of extraction and analysis of VOCs from different plant species cited in Section 5.1 and Section 5.2 below.

### 5.1. Extraction of VOCs

To isolate them, plant and flower volatiles can be obtained by extraction using solvents and distillation (by water or steam) [75,76,95]. Traditional methods include distillation (hydrodistillation) and extraction with organic solvents. More recent methods have been developed, like microwaves used as the heat source to assist the extraction or supercritical fluid extraction, and the advantages and limitations of these methods have been reviewed in depth [96]. Fast on-site (mobile) detection technologies have also been developed, which changes the approach from “sample to the lab” to “lab to the sample” [97]. Finally, flower volatiles can be obtained from the airspace surrounding the living or excised flower (headspace techniques) [98]. Both solvent extraction and water or steam distillation are time-consuming methods, and both are susceptible to artifacts, the former primarily by isolating non-volatile substances from the tissue, the latter primarily by heat-induced rearrangements [96]. Also, solvent extraction requires the use of large amounts of solvent, and distillation uses a relatively large amount of sample [77]. In addition, soft extraction methods, such as headspace methods, are preferred due to the specific requirements of botanical research. Headspace techniques provide a more realistic picture of the volatile profile than that obtained with traditional solvent extraction or distillation of flower tissue, so they are increasingly used by researchers [78]. Therefore, in the paragraph below, we will focus on headspace techniques.

Headspace sampling is a non-destructive method of collecting volatiles; it involves trapping volatiles found in the gas space above the sample on an absorbent matrix [72]. Headspace sampling techniques include static and dynamic sampling modes. For dynamic sampling modes, a continuous flow of air passes through the sample container as the carrier gas. In effect, while the analytes are trapped on the adsorbents, the carrier gas flows through or is discharged from the container, allowing sufficient amounts of volatiles to be collected. For static modes, the live or cut flower is enclosed in a container, and the emitted volatiles are trapped on an ab/adsorbent. The air surrounding the plant remains static. The volatiles are enriched on the ab/adsorbent matrix without sampling impurities from a continuous air stream that can interfere with the detection of low-abundant VOCs [99].

An important advance in static headspace sampling was the development of solid-phase microextraction (SPME) [38]. This technique was introduced in 1990 by Arthur and Pawliszyn to analyze pollutants in water [100]. Since then, its field of application has developed enormously, particularly in the analysis of the odorant components of many flowers and other plant materials [77,79,80,101]. Indeed, it is a technique that presents several advantages, such as the reduction of the extraction time, the absence of the use of organic solvents, the possibility of automation, and the facility of coupling with gas chromatography (GC) [81]. The SPME allows, with a single device, the collection, concentration, derivatization (if necessary), and introduction of the VOCs into the injector in a continuous and uninterrupted process, which considerably reduces the sample preparation time [82]. It is also widely used due to its simplicity, relatively low sample uptake, low cost, selectivity, and sensitivity [80]. SPME is based on the ab/adsorption of compounds onto a fiber coated with a polymer, also called the extraction phase. The fiber is placed inside a removable hollow needle; volatile substances can be sampled by inserting the needle through the septum of a container and pushing the plunger to expose the fiber [102]. The analytes will be gradually ab/adsorbed by the extraction phase. After a sufficient period of time called equilibration time, a partitioning equilibrium is established between the solid phase constituted by the fiber and the gas or liquid phase. The fiber is then retracted into the needle and removed from the sample. For extraction, the fiber can be directly immersed in the liquid, in the presence of a liquid sample, in which case it is called direct immersion (DI-SPME), or it can be exposed to the vapor phase in the presence of a solid, liquid, or gaseous sample, in which case it is called headspace-SPME (HS-SPME) [103]. The analytes are subsequently desorbed for analysis. Desorption can be performed thermally or by the use of organic solvents. However, the broad peak of the organic solvent can be a potential interference with the volatile analytes in the chromatographic analysis when a solvent is used for desorption. Thus, the release of analytes by thermal desorption requires the use of ab/adsorbent fibers with good thermal stability, which provides a higher desorption efficiency than solvent elution and avoids sample dilution [82].

SPME fibers can be reused about 100 times, and several polymers are currently available. There are single-phase sorbents such as polydimethylsiloxane (PDMS) and polyacrylate (PA) and mixed-phase sorbents, the best known of which are carboxene/polydimethylsiloxane (CAR/PDMS), polydimethylsiloxane/divinylbenzene (PDMS/DVB), and divinylbenzene/carboxene/polydimethylsiloxane (DVB/CAR/PDMS) [79,81,104]. PDMS is the most widely used; it is a rubbery liquid with high viscosity. PA, on the other hand, is a crystalline solid coating that turns into a liquid at desorption temperatures. Both of these single-phase sorbents extract analytes by absorption, whereas for mixed-phase sorbents, the primary extraction phase is a porous solid, which extracts analytes by adsorption [105]. The analytes initially bind to the surface of the coating regardless of its nature. Whether they migrate to the bulk of the coating or remain on its surface depends on the magnitude of the diffusion coefficient of an analyte in the coating. In PDMS, the diffusion coefficients of analytes are close to those of organic solvents, and diffusion is relatively fast. In PA, even if the diffusion coefficients of the analytes are lower than in PDMS, they remain sufficiently important for absorption to be the main extraction mechanism. For mixed phases, the diffusion coefficients of analytes in divinylbenzene and carboxene are so low that they remain essentially on the surface of the coating [105]. Because the polymers also have different polarities, by carefully selecting the polarity and thickness of the fiber coating, compounds of different polarity and volatility can be extracted. This is because polar compounds are sorbed onto polar fibers and non-polar compounds onto non-polar fibers, so it is possible to sample analytes of different volatilities, ranging from high-boiling or semi-volatile compounds to highly volatile compounds [106]. The choice of the nature of the fiber is, therefore, very important to perform an extraction by SPME [83].

Considering that SPME is a technique based on physicochemical equilibration processes between the sample and the headspace and between the headspace and the fiber coating, apart from the nature of the fiber, several other parameters will have to be optimized in order to ensure its successful use [84,85,86,87,107]. Indeed, parameters such as extraction temperature and duration, equilibration time, desorption time, and agitation can also affect the SPME method [108]. Since the success of a good SPME extraction depends on all these factors, this technique is not considered a quantitative method. Indeed, SPME is better suited to studies of VOC mixtures or to the determination of the presence and/or absence of certain compounds. Quantification of VOCs with SPME is possible when careful control of sampling parameters is achieved and appropriate mixtures of standards for calibration are used. However, it is very difficult when a wide range of compounds with different distribution constants are present [102].

### 5.2. Analysis of VOCs

The most widely used technique for the analysis of volatile or semi-volatile compounds is gas chromatography-mass spectrometry (GC-MS) [52,89,90]. It is a method that combines the performance of gas chromatography, which allows the separation of compounds, and the performance of mass spectrometry, which allows the detection and identification of compounds according to their mass-to-charge ratio (*m*/*z*).

The gas chromatograph consists of an injection system, a column placed in an oven that is made up of a stationary phase in which the molecules of the mixture to be analyzed are separated, and a detection system. The analytes are injected into the apparatus and then carried into the column by a flow of inert carrier gas called the mobile phase. In the column, the analytes, having different travel speeds, are separated in time. The travel time of each analyte depends on its volatility and the interactions between the stationary phase and the analyte [109]. Since the oven can regulate the temperatures applied to the column, a decrease in temperature increases the interaction between the analytes and the stationary phase and leads to a longer retention time in the apparatus. Most often, a temperature gradient is used to separate molecules in a complex mixture [91,92]. The analytes are detected as they leave the column and are characterized by the retention time corresponding to the time elapsed between the injection of the analyte and the moment of its arrival at the detector. In GC, the analytes can be injected in different ways. The injection can be carried out in split mode (with flow division), which is used for concentrated samples; in splitless mode (without flow division), which is used for analytes in dilute solution; and in on-column mode (in the column), which is used for thermolabile compounds or to inject a large volume. In this case, there is no vaporization step; the sample is injected directly into the column [110]. The coupling of HS-SPME to GC allows the introduction of analytes by thermal desorption. In the case where VOCs are desorbed by a thermal desorption unit (TDU), two steps are necessary before injection. The analytes released from the ab/adsorbent by heat are first concentrated in a cold trap and then transferred from the cold chamber to the chromatographic column via a transfer line [102]. After injection, separation of the compounds takes place in the column. This separation is generally carried out on capillary columns consisting of a tube of fused silica whose internal wall is covered with the stationary phase characterized by the chemical functions grafted on the silica [93]. The main stationary phases can be apolar, mainly composed of silicone polymers derived from polydimethylsiloxane, polar based on polyethylene glycol (PEG), or chiral containing hydrophobic cyclodextrins to separate chiral molecules.

There are different types of detectors, but the mass spectrometer now tends to supplant all others because it is the only one that provides structural information on chromatographically separated compounds. There are many types of mass spectrometers that all have three elements in common: a source, an analyzer, and a detector [111]. However, for most standard benchtop GC-MS instruments, compounds exiting the GC column are ionized by electron impact (EI), and the resulting ions are selected based on their mass-to-charge ratio (*m*/*z*) using a quadrupole analyzer [112]. The detector leads to the fragmentation spectrum of each compound. Total ion chromatograms are obtained, and they provide information on the retention time of each compound as well as their mass spectrum containing the *m*/*z* ratios of the detected ions.

In order to identify volatile compounds, suggestions can be obtained from popular mass spectra libraries such as the Wiley and NIST mass spectra databases [88]. Additional confidence in VOC identification can be obtained by comparing compound retention indices calculated from a mixture of alkanes with those in online databases [94]. Retention indices are used to convert retention times into system-independent constants. The concept was first described in the 1950s by the Swiss chemist Ervin Kovats [113]. Finally, a complete identification is achieved by comparing the retention times and mass spectra of compounds with those of available authentic standards [72].

## 6. Conclusions and Future Perspectives

Flowers produce complex volatile mixtures that constitute a highly diversified group of signaling molecules with important ecological functions such as attracting pollinators, defending the plant against biotic and abiotic attacks, and allowing the plant to communicate with its environment. Each flower has a unique fragrance due to the relative quantities and diversity of VOCs as well as the interactions between these compounds. Thus, it has been reported that floral chemical profiles vary in many species, both within the same individual, between individuals, and between populations. Thus, it has been reported that floral chemical profiles vary in many species, both within and between individuals and populations. This intra- and interspecific variation has led scientists to conduct numerous research studies. Among the factors identified, pollination plays an important role in the variation of chemical profiles between conspecific populations. In addition to pollination, florivores and herbivores, flower age, habitat, and environment are important factors in the variation of VOCs. Because of this variation, identifying the authentic profile of emitted volatiles can be difficult. As a result, researchers must first decide whether they are interested in the qualitative profile of the volatiles released at a particular time or whether they want to measure quantitative changes in VOC emissions requiring adequate instrumentation to collect and analyze these compounds. The preferred methods for collecting floral odor are headspace methods, which are easy to apply and provide the ability to isolate volatile compounds from either a live flower or a cut flower depending on the type of study being conducted. The compounds are then analyzed and identified thanks to the GC-MS, which is a technique that has evolved a lot since its creation with the launch of very powerful devices including different modules according to the type of application.

However, some air pollutants are known to have an impact on the VOCs emitted by flowers and the pollinators population. A better comprehension of the influence of different pollutants on plant-plant, plant-pollinator, plant-pathogen, or plant-florivore interactions and of the mechanism involved would provide new insights into the impacts of the anthropogenic air pollution on the natural ecosystems in conjunction with environmental changes and global warming.

## Figures and Tables

**Table 1:** Some examples of VOCs with their associated ecological bioactivity (not exhaustive).

Compounds	Chemical Structure	Fam. ^1^	Examples of Bioactivity: Pollination/Defense/Communication	Ref.
linalool	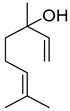	M	Moth-pollinated *Clarkia breweri* flowers emission	[18]
			Pollination by butterflies or nocturnal moths	[19,20,21]
			Aphids repulsion	[14,22]
geraniol	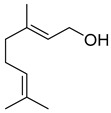	M	Dominent messenger in chemical communication among bees, abundant emission from *Coffea arabica* flowers	[23,24,25]
(*E*)-β-ocimene	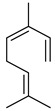	M	Systematic release by tobacco plants damaged by herbivore *Heliothis virescens*	[26]
α-pinene	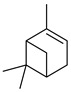	M	Response to increasing drought stress: *Ipomopsis aggregata* flower emission highest percentage of the scent mixture when intermediate soil moisture	[27]
(*E*,*E*)-α-farnesene	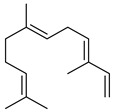	S	Response to increasing drought stress: *Ipomopsis aggregata* flower emission highest percentage of the scent mixture when intense soil drought	[27]
(*E*)-α-bergamotene (α-*trans*-bergamotene)	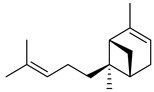	S	Herbivore induced sesquiterpene	[28]
β-caryophyllene	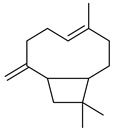	S	Systematic release by tobacco plants damaged by herbivore *Heliothis virescens*	[26]
			Emission by apple trees infected by *Candidatus* Phytoplasma mali	[29]
			*Arabidopsis* *thaliana* floral defense against microbial pathogen	[30]
methyl benzoate	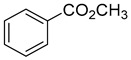	P/B	Snapdragon *Antirrhinum majus* floral VOC with negative allelopathic effects on *Arabidopsis thaliana*.	[31]
methyl salicylate and other salicylates	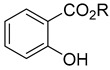 R=CH_3_, other	P/B	Released at a significantly higher rate from aphid-infested plants	[32,33,34]
(*Z*)-3-hexen-1-ol	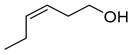	Al	Can initiate a subset of regulatory steps and downstream metabolites to initiate defense responses in maize. Function as indirect plant defense mediating plant-plant signaling and intraplant information transfer.	[35,36]
(*E*) and (*Z*) 3-hexenal	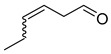	A	Implicated in plants protection against insect herbivore attack. Numerous activities in plants pathogen resistance	[33,37]
indoles	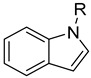 R=CH_3_: skatole	O	*Stapelia gigantea* or *Wurmbea elatior* flower emission for coprophagous insects attraction	[38,39]

^1^ Family of compounds: M = Monoterpene, S = Sesquiterpene, P/B = Phenylpropanoids/Benzenoids, Al = Alcohol, A = Aldehyde, O = Other.

**Table 2:** Methods of extraction and analysis of VOCs from different plant species cited in Section 5.1 and Section 5.2.

Methods of Extraction of VOCs	Analytical Methods of VOCs	Plants	References (Year)
HS-SPME	GC-MS	Tropical flowers	[38] (2023) (review)
Steam distillation	GC-MS	*Prunella vulgaris* whole plant	[75] (2021)
Simultaneous steam distillation and solvent extraction	GC-MS	*Coccinia abyssinica* (Anchote) leaf and tuber	[76] (2022)
Hydrodistillation and HS-SPME	GC-MS	*Thymus mastichina* flowers and leaves	[77] (2006)
HS-SPME	GC-MS	*Hedychium coronarium* (ginger lily) flowers	[78] (2011)
HS-SPME	GC-MS	*Narcissus tazetta* var. chinensis (chinese daffodil) flowers	[79] (2007)
HS-SPME	GC-MS	*Salvia mirzayanii* flowers, leaves and stems	[80] (2015)
HS-SPME	GC-FID	*Smallanthus sonchifolius* leaves	[81] (2005)
HS-SPME	GC-MS, GC-FID and GC-ECD	*Brugmansia suaveolens* flowers	[82] (2008) (review)
HS-SPME and S-HS	GC-MS	*Rosmarinus officinalis, Salvia officinalis, Thymus vulgaris, Valeriana officinalis*	[83] (2000)
HS-SPME	GI-FID and GC-MS	*Capsicum frutescens* and other *Capsicum* chili peppers	[84] (2011)
HS-SPME	GC-MS	*Osmanthus fragrans* var. latifolius and *Osmanthus fragrans* var. *thunbergii* flowers	[85] (2004)
HS-SPME	GC-HRMS	Brassicaceae vegetables	[86] (2018)
HS-SPME	GC-MS	*Tillandsia* species	[67,87,88] (2021, 2022)
Hydrodistillation and HS-SPME	GC-MS	*Galium verum* aerial parts (leaves and flowers)	[89] (2020)
HS-SPME	GC-MS	*Ceratonia siliqua* (carob trees) flowers	[90] (2006)
HS-SPME	GC-MS	*Silene latifolia* flowers	[47] (2005)
HS-SPME	GC-MS	*Chrysanthemum* cultivar flowers	[91] (2015)
HS-SPME and HS-DTD	GC-MS	*Rosa odorata* and *Rosa chinensis*	[92] (2020)
HS-SPME	GC-MS	*Michelia alba* flowers	[93] (2002)
HS-SPME	GC-MS	*Brunfelsia pauciflora* and *Brunfelsia australis*	[94] (2006)

DTD: direct thermal desorption; ECD: electron capture detection; FID: flame ionization detection; GC: gas chromatography; HRMS: high-resolution mass spectrometry; HS: headspace; MS: mass spectrometry; S-HS: static headspace; SPME: solid phase microextraction.

## Data Availability

Not applicable.
